# Association of adverse cardiovascular events with gabapentin and pregabalin among patients with fibromyalgia

**DOI:** 10.1371/journal.pone.0307515

**Published:** 2024-07-26

**Authors:** Yiheng Pan, Robert P. Blankfield, David C. Kaelber, Rong Xu

**Affiliations:** 1 Computer and Data Science Department, Case Western Reserve University Case School of Engineering, Cleveland, OH, United States of America; 2 Center for Artificial Intelligence in Drug Discovery, Case Western Reserve University School of Medicine, Cleveland, OH, United States of America; 3 Department of Family Medicine, Case Western Reserve University School of Medicine, Cleveland, OH, United States of America; 4 The Center for Clinical Informatics Research and Education, The Metro Health System, Cleveland, OH, United States of America; University of Würzburg, GERMANY

## Abstract

**Objective:**

Fibromyalgia, a chronic pain disorder, impacts approximately 2% of adults in the US. Gabapentin and pregabalin are common treatments to manage fibromyalgia-related pain. Our recent study showed the risk of adverse cardiovascular events increased in diabetic neuropathy patients who were prescribed gabapentin or pregabalin. Here, we investigated whether the prescription of gabapentin or pregabalin has similar cardiovascular risk in patients with fibromyalgia.

**Methods:**

This retrospective cohort study leveraged electronic health records from 64 US healthcare organizations with 112 million patients. The study population included 105,602 patients first diagnosed with fibromyalgia and followed by a prescription of gabapentin, pregabalin, or other FDA-approved drugs for treating fibromyalgia from 2010 to 2019. Outcomes were deep venous thrombosis (DVT), myocardial infarcts (MI), peripheral vascular disease (PVD), strokes, heart failure, and pulmonary embolism (PE). In propensity-score-matched cohorts, 1-year and 5-year hazard ratios (HRs) were computed with their respective 95% confidence intervals (CIs). Additionally, we conducted sensitivity analyses on the subpopulations without other possible indications.

**Results:**

For 5-year follow-up, gabapentin increased the risk of PVD (HR = 1.46, 95% CI = 1.17–1.80), MI (HR = 1.31, 95% CI = 1.03–1.66), heart failure (HR = 1.27, 95% CI = 1.10–1.48), DVT (HR = 1.80, 95% CI = 1.33–2.44), and PE (HR = 2.23, 95% CI = 1.62–3.07). Pregabalin increased the risk of DVT (HR = 1.49, 95% CI = 1.01–2.20), and PE (HR = 2.24, 95% CI = 1.43–3.50). For 1-year follow-up, gabapentin increased the risk of PVD (HR = 1.32, 95% CI = 1.11–1.57), DVT (HR = 1.35, 95% CI = 1.09–1.68), and PE (HR = 1.36, 95% CI = 1.17–1.57). Pregabalin increased the risk of PVD (HR = 1.32, 95% CI = 1.06–1.63) and PE (HR = 1.25, 95% CI = 1.03–1.52). Sensitivity analyses showed similar trends.

**Conclusion:**

In fibromyalgia patients, the prescription of gabapentin and pregabalin moderately increased the risk of several adverse cardiovascular events. This risk, together with benefits and other adverse reactions, should be considered when prescribing these medications for fibromyalgia patients.

## Introduction

Fibromyalgia is characterized by fatigue, diffuse muscle pain, poor sleep and trigger points of tenderness[1, 2]. The prevalence of fibromyalgia is approximately 2% of the U.S. population, but it more often affects women than men [3]. The management of fibromyalgia includes both pharmacological and nonpharmacological treatments. Medications with the best evidence to treat fibromyalgia include tricyclic antidepressants such as amitriptyline and nortriptyline; muscle relaxants such as cyclobenzaprine; serotonin-norepinephrine reuptake inhibitors; and gabapentinoids [4]. The treatment of fibromyalgia was approved by the U.S. Food and Drug Administration (FDA) for three medications: duloxetine (Cymbalta), pregabalin (Lyrica), and milnacipran (Savella) [5]. Despite not being officially approved, gabapentin is frequently prescribed to manage fibromyalgia [6].

Recently, our group identified that prescribing gabapentin and pregabalin to diabetic neuropathy patients may elevate their cardiovascular risk [7]. However, the association between the prescription of these medications and adverse cardiovascular events in patients with fibromyalgia is uncertain.

We leveraged a large-scale national electronic health record (EHR) system to conduct a cohort study. The goal was to determine whether the prescription of gabapentin and pregabalin in patients with fibromyalgia increases the risk of adverse cardiovascular events, including peripheral vascular disease, strokes, myocardial infarcts, heart failure, deep venous thrombosis, and pulmonary embolism.

## Materials and methods

### Database description

This retrospective cohort study used TriNetX platform (the US Collaborative Network) to derive our aggregated and deidentified EHR data including 112 million patients across 64 healthcare organizations throughout the United States [8]. We accessed the dataset on May 30^th^, 2024. This dataset encompasses a wide range of varied geographic regions, age groups, races and ethnicities, economic statuses, and types of health insurance. We previously used TriNetX platform to conduct retrospective cohort studies to investigate drug effects [9–22].

### Ethics approval and informed consent

The TriNetX platform aggregates and HIPAA de-identifies data contributed from the electronic health records of participating healthcare organizations. The TriNetX platform also only reports population level results (no access to individual patient data) and uses statistical “blurring”, reporting all population level counts between 1 and 10 as 10. Based on the de-identification methods used by TriNetX, as per HIPAA privacy and security rules [23], TriNetX sought and obtained expert attestation that TriNetX data is HIPAA de-identified (see attached for official expert TriNetX HIPAA deidentification attestation). Because the data in the TriNetX platform is HIPAA de-identified, and therefore, “by definition” is deemed to allow no access to protected health information (and therefore no risk of protected health information disclosure), Institutional Review Boards (IRBs) have no jurisdiction of studies using HIPAA de-identified data (IRB factsheet for print 4-23-04.p65 (nih.gov) [24]). Therefore, IRB approved was neither sought nor obtained for this study. This study follows with Strengthening the Reporting of Observational Studies in Epidemiology (STROBE) statement [25].

### Study population

The study population involved 105,602 patients who received an initial ICD diagnosis of fibromyalgia (M79.7) and were subsequently prescribed gabapentin, pregabalin, duloxetine, or milnacipran in their electronic health records from 2010–2019. There are three distinct groups in the study population: (1) an exposure group included fibromyalgia patients who had a prescription of gabapentin and were not prescribed pregabalin, duloxetine, or milnacipran; (2) another exposure group included fibromyalgia patients who had a prescription of pregabalin but not prescribed gabapentin, duloxetine or milnacipran; and (3) a comparison group included fibromyalgia patients who had a prescription of either duloxetine or milnacipran (other FDA-approved drugs) and were not prescribed gabapentin or pregabalin. To investigate the results of 5-year follow-up, we created the cohorts by the added criterion that they received repeated prescriptions for the same drug recorded in EHR at least 3 years following their initial prescription (Fig 2).

#### Statistical analysis

The Kaplan-Meier Analysis approximates the probability of the outcome within specific time intervals with a daily time interval being employed for this analysis. Patients are censored when they no longer contribute information in the analysis. The assessment of proportional hazard was evaluated using the generalized Schoenfeld approach. The hazard ratio and corresponding confidence intervals, along with the proportionality test, were calculated using version 3.2–3 of the Survival package in R.

### Outcome measures

The outcomes were the presence of cardiovascular disease diagnosis codes (ICD-10), including I73.9 (peripheral vascular disease), I63 (stroke), I21 (myocardial infarction), I50 (heart failure), I82.40 (deep venous thrombosis), and I26 (pulmonary embolism). The comprehensive list of outcomes, their corresponding standard names, identifiers, and data formats is presented in S1 Table in S1 File.

### Covariates

The covariates included demographic information (sex, race, age, and ethnicity), comorbidities of fibromyalgia, specific risk factors for each adverse cardiovascular event, adverse socioeconomic circumstances, other potential drug purposes including those approved by the FDA and those beyond approval, pain-related medications, diabetic medications, cardiovascular medications, antihypertensive medications. The full list of covariates is presented in S2 Table in S1 File.

The Exposure groups and Comparison groups were balanced using propensity scores at a 1:1 ratio, employing the nearest neighbor greedy matching algorithm based on the previously described covariates. To obtain the risk of each adverse cardiovascular event in these groups, a Kaplan–Meier analysis was conducted. For each group, the initial prescription of the medication served as the index event. The covariates were measured at any time point on or before the time of the index event. Patient outcomes were assessed over two distinct follow-ups: one-year and five-year beginning from the date of initial drug prescription. We calculated hazard ratios, along with their 95% confidence intervals (CIs), as well as corresponding p-values. Significance was determined at a threshold of p-value < 0.05, with two-sided testing.

**Fig 1:**
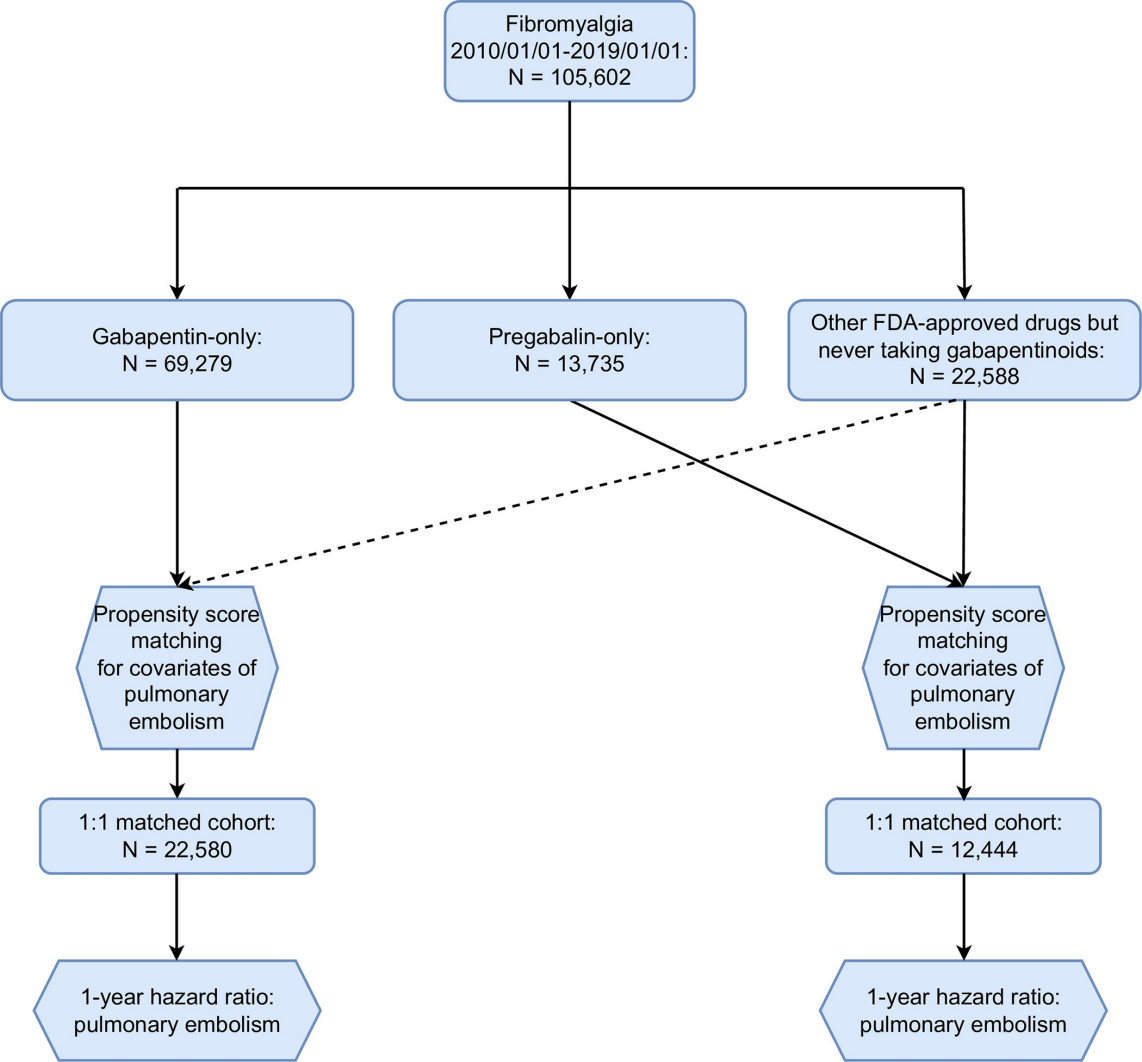
Flowchart of patient selection and analysis of the 1-year risk of pulmonary embolism in TriNetX.

**Fig 2:**
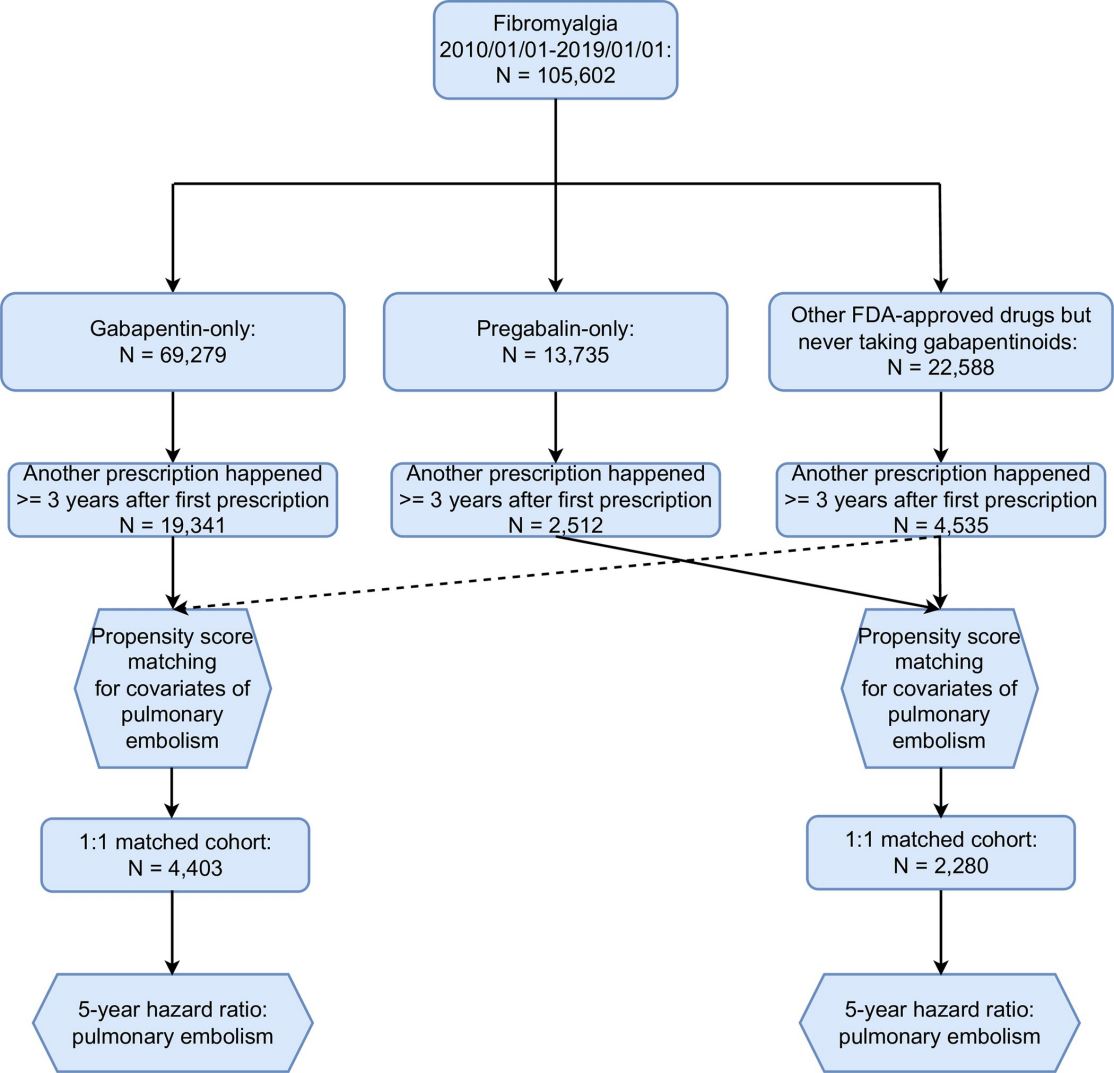
Flowchart of patient selection and analysis of the 5-year risk of pulmonary embolism in TriNetX.

Fig 1 presents the flowchart detailing the process of the patient selection and analysis in TriNetX examining the risk of pulmonary embolism within 1 year, and Fig 2 shows the flowchart of the selection of patients with repeated prescription and analysis examining the risk of pulmonary embolism within 5 years.

### Sensitivity analyses

In the main analyses, we used propensity score matching for other potential indications for gabapentinoids. To evaluate the soundness of the results of main analyses, we conducted sensitivity analyses for the subpopulation without any diagnosis of other potential indications, including diabetic neuropathy, seizure, postherpetic neuralgia, neuropathic pain, or restless leg syndrome.

## Results

### (1) Patient characteristics

Table 1 displays the demographic and risk factor characteristics of fibromyalgia patients: those who had repeated prescriptions of gabapentin three years after their initial prescription of gabapentin, and those who had repeated prescriptions of comparison drugs three years after their initial prescription of the comparison drugs. The table presents data both before and after using propensity-score matching for pulmonary embolism-related covariates. S3 Table in S1 File presents the comprehensive covariate characteristics before and after matching. Before matching, individuals in the gabapentin cohort were, on average, older at 55.7 years compared to 53.2 years in the comparison cohort, and they exhibited a significantly greater prevalence of comorbid conditions and worse socioeconomic health factors. After the propensity-score matching, the two cohorts achieved a balanced state, resulting in 4,403 patients each in the matched gabapentin and comparison groups. Details on pregabalin data and patient characteristics over short-term follow-up can be found in S4-S6 Tables in S1 File.

**Table 1:** Characteristics of fibromyalgia patients with repeated prescription of gabapentin and those with repeated prescription of comparison drugs before and after applying propensity-score matching for pulmonary embolism-related covariates.

Characteristics	Before propensity-score matching			After propensity-score matching		
	Cohort, No. (%)			Cohort, No. (%)		
	Gabapentin cohort	Comparison cohort	SMD	Gabapentin cohort	Comparison cohort	SMD
Total no	19,019	4,412		4,403	4,403	
Age at Index	55.7 ± 14.6	53.2 ± 13.8	0.17*	53.4 ± 14.6	53.3 ± 13.8	0.01
Female	74.4	84.8	0.26*	85.0	84.7	0.01
White	68.7	76.7	0.18*	76.6	76.7	<0.001
Black or African American	15.8	7.8	0.25*	7.7	7.8	<0.001
Hispanic or Latino	7.3	5.2	0.09	5.3	5.2	<0.001
Other Race	2.1	1.7	0.03	1.9	1.7	0.02
Asian	1.4	0.7	0.06	0.8	0.7	<0.001
Neoplasms	37.1	25.3	0.26*	24.6	25.3	0.02
Overweight and obesity	26.5	23.7	0.06	23.6	23.6	<0.001
Migraine	15.8	17.7	0.05	17.5	17.7	<0.001
Diabetes mellitus	26.8	16.4	0.26*	16.7	16.4	0.01
Urinary tract infection, site not specified	20.0	14.2	0.15*	14.8	14.2	0.02
Obstructive sleep apnea	12.9	11.6	0.04	11.0	11.6	0.02
Irritable bowel syndrome	8.0	9.3	0.05	8.9	9.3	<0.001
Pain	11.7	7.7	0.13*	7.4	7.7	<0.001
Coronary artery disease	12.3	6.6	0.20*	7.2	6.6	0.03
Persons with potential health hazards related to socioeconomic and psychosocial circumstances	4.3	3.6	0.04	4.2	3.6	0.03
Chronic fatigue, unspecified	2.5	4.3	0.10*	4.1	4.2	0.01
Restless legs syndrome	5.7	3.9	0.09	3.6	3.9	0.01
Heart failure	7.4	3.4	0.18*	3.4	3.4	<0.001
Panic disorder	3.6	3.0	0.04	3.2	3.0	0.01
Tobacco use	3.8	2.9	0.05	3.2	3.0	0.01
Neuralgia and neuritis, unspecified	6.6	2.2	0.21*	2.6	2.2	0.03
Diabetic neuropathy	6.0	1.9	0.21*	2.3	1.9	0.03
Epilepsy and recurrent seizures	3.1	2.0	0.07	2.1	2.0	0.01
Use of NSAID	1.6	1.7	0.01	1.9	1.7	0.01
Peripheral vascular disease	4.8	1.8	0.17*	1.8	1.8	0.01
Stroke	3.9	1.8	0.12*	1.8	1.8	0.01
Alcohol abuse	4.0	1.2	0.18*	1.2	1.2	<0.001
Pulmonary embolism	2.3	0.9	0.11*	1.0	0.9	0.01
Hypoglycemia	1.9	1.0	0.08	1.0	1.0	<0.001
End stage renal disease	1.3	0.3	0.11*	0.2	0.3	0.01
Other postherpetic nervous system involvement	0.7	0.2	0.06	0.2	0.3	<0.001

NSAID: non-steroidal anti-inflammatory drugs

* An SMD (Standardized Mean Difference) exceeding 0.1, a threshold commonly advised for indicating an imbalance

### (2) 5-year outcomes for patients with fibromyalgia who had repeated prescriptions of gabapentin or pregabalin versus those who had repeated prescriptions of comparison drugs

**Fig 3:**
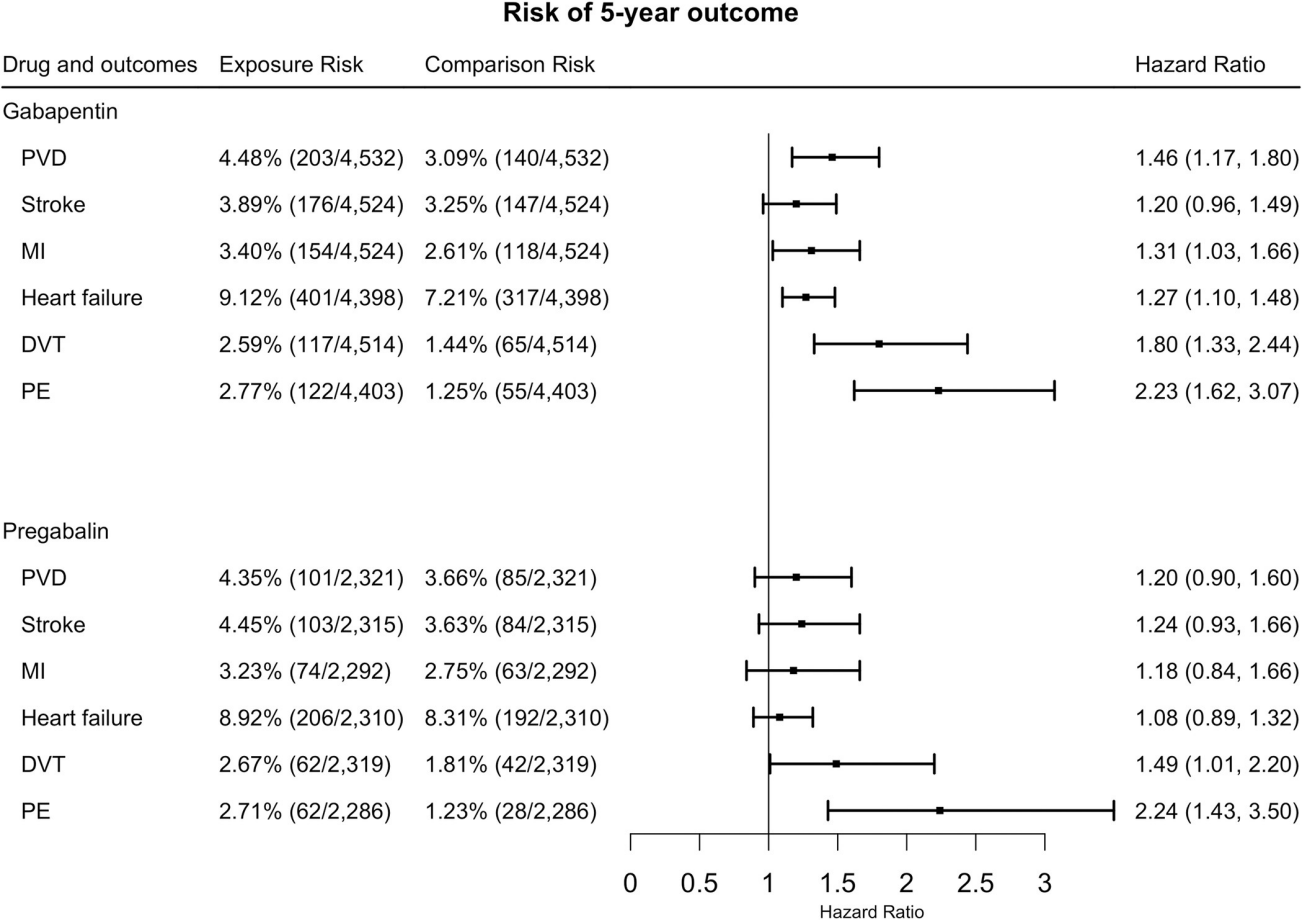
The hazard ratios for 5-year cardiovascular risk between exposures and comparisons after propensity score matching. (PVD: peripheral vascular disease, PE: pulmonary embolism, MI: myocardial infarction, DVT: deep venous thrombosis).

For 5-year follow-up, gabapentin increased the risk of PVD (HR = 1.46, 95% CI = 1.17–1.80), MI (HR = 1.31, 95% CI = 1.03–1.66), heart failure (HR = 1.27, 95% CI = 1.10–1.48), DVT (HR = 1.80, 95% CI = 1.33–2.44), and PE (HR = 2.23, 95% CI = 1.62–3.07). No significant differences were detected for stroke (HR = 1.20, 95% CI = 0.96–1.49), (Fig 3).

Pregabalin increased the risk of DVT (HR = 1.49, 95% CI = 1.01–2.20), and PE (HR = 2.24, 95% CI = 1.43–3.50). There was no significant difference observed for PVD (HR = 1.20, 95% CI = 0.90–1.60), and stroke (HR = 1.24, 95% CI = 0.93–1.66) MI (HR = 1.18, 95% CI = 0.84–1.66) or heart failure (HR = 1.08, 95% CI = 0.89–1.32) (Fig 3).

### (3) 1-year outcomes for patients with fibromyalgia prescribed with gabapentin or pregabalin versus those prescribed with other drugs

**Fig 4:**
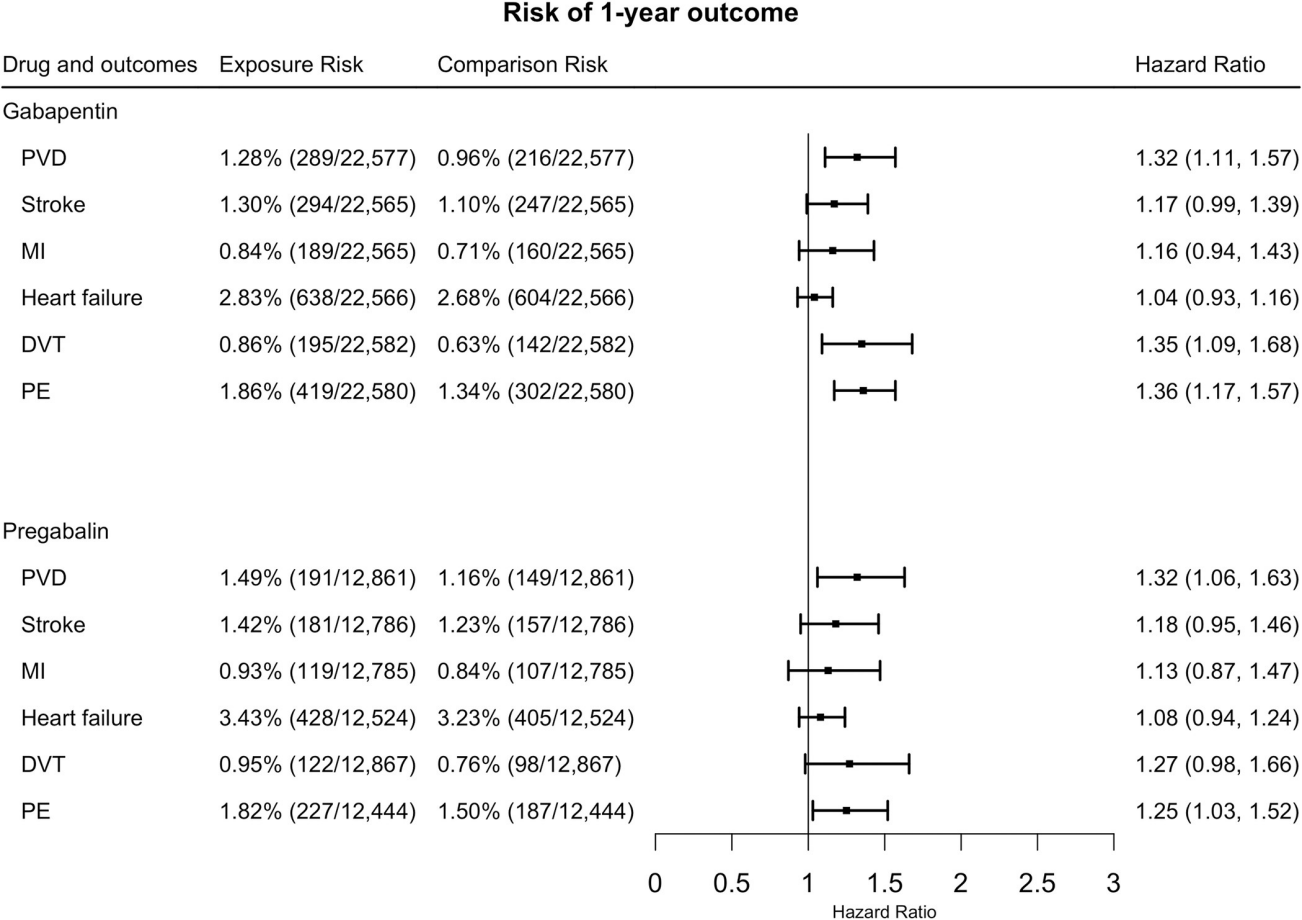
The hazard ratios for 1-year cardiovascular risk between exposures and comparisons after propensity score matching. (PVD: peripheral vascular disease, PE: pulmonary embolism, MI: myocardial infarction, DVT: deep venous thrombosis).

For 1-year follow-up, gabapentin increased the risk of PVD (HR = 1.32, 95% CI = 1.11–1.57), DVT (HR = 1.35, 95% CI = 1.09–1.68), and PE (HR = 1.36, 95% CI = 1.17–1.57). No significant differences were detected for stroke (HR = 1.17, 95% CI = 0.99–1.39) or MI (HR = 1.16, 95% CI = 0.94–1.43), or heart failure (HR = 1.04, 95% CI = 0.93–1.16) (Fig 4).

Pregabalin increased the risk of PVD (HR = 1.32, 95% CI = 1.06–1.63) and PE (HR = 1.25, 95% CI = 1.03–1.52). There were no significant differences suggested for stroke (HR = 1.18, 95% CI = 0.95–1.46), MI (HR = 1.13, 95% CI = 0.87–1.47), or heart failure (HR = 1.08, 95% CI = 0.94–1.24), or DVT (HR = 1.27, 95% CI = 0.98–1.66) (Fig 4).

### (4) 5-year outcomes for patients with fibromyalgia who had repeated prescriptions of gabapentin or pregabalin versus those who had repeated prescriptions of comparison drugs in sensitivity analyses

**Fig 5:**
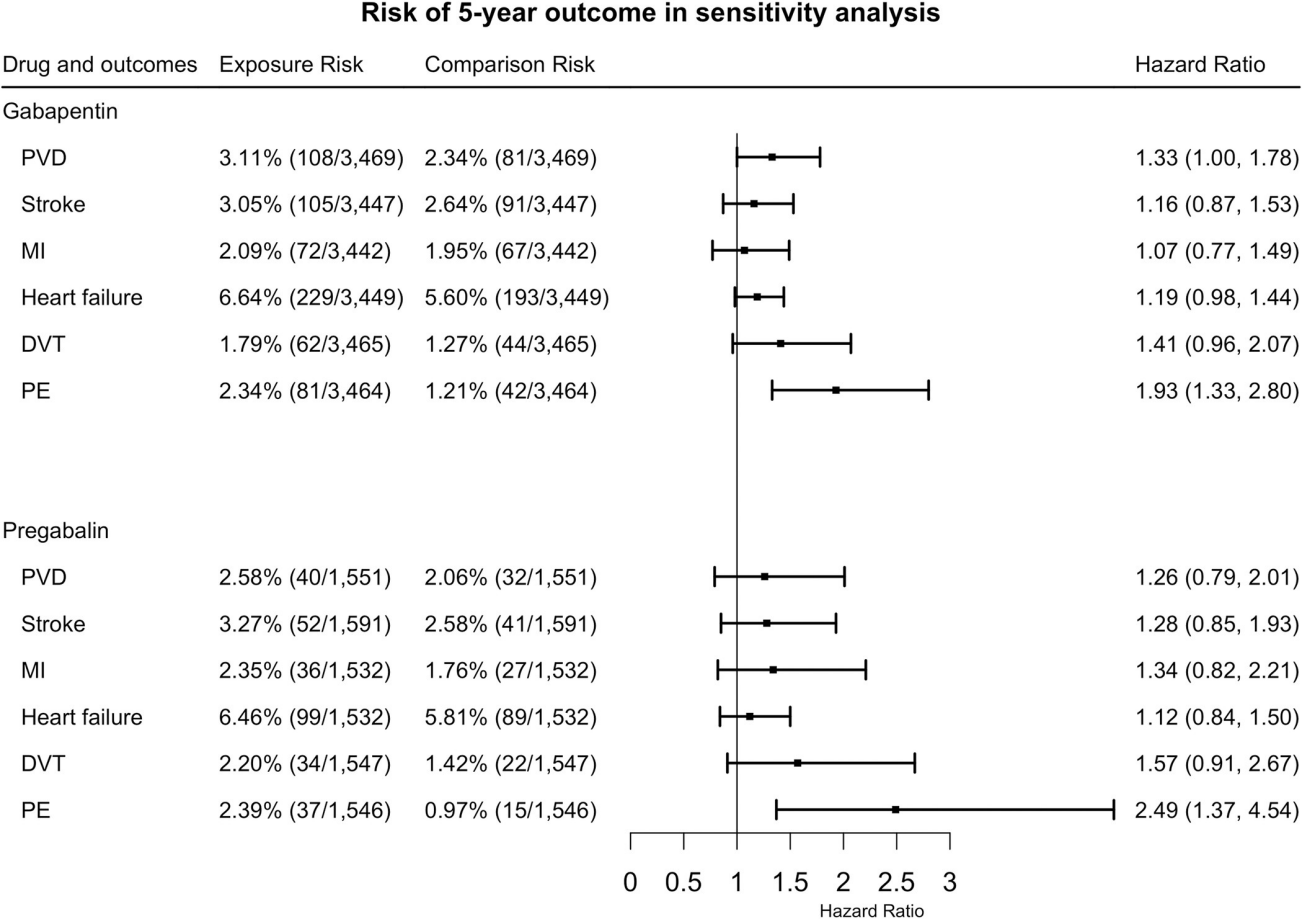
The hazard ratios for 5-year cardiovascular risk between exposures and comparisons after propensity score matching among the subpopulation who had no other possible indications. (PVD: peripheral vascular disease, PE: pulmonary embolism, MI: myocardial infarction, DVT: deep venous thrombosis).

In sensitivity analyses of subpopulations without other possible indications, gabapentin increased the 5-year risk of PVD (HR = 1.33, 95% CI = 1.00–1.78), PE (HR = 1.93, 95% CI = 1.33–2.80). No significant increase in risk was detected for stroke (HR = 1.16, 95% CI = 0.87–1.53), heart failure (HR = 1.19, 95% CI = 0.98–1.44), DVT (HR = 1.41, 95% CI = 0.96–2.07), or MI (HR = 1.07, 95% CI = 0.77–1.49) (Fig 5).

Pregabalin increased the 5-year risk of PE (HR = 2.49, 95% CI = 1.37–4.54). No significant differences were detected for PVD (HR = 1.26, 95% CI = 0.79–2.01), stroke (HR = 1.28, 95% CI = 0.85–1.93), heart failure (HR = 1.12, 95% CI = 0.84–1.50), or MI (HR = 1.34, 95% CI = 0.82–2.21), or DVT (HR = 1.57, 95% CI = 0.91–2.67) (Fig 5).

### (5) 1-year outcomes for patients with fibromyalgia prescribed with gabapentin or pregabalin versus those prescribed with other drugs in sensitivity analyses

**Fig 6:**
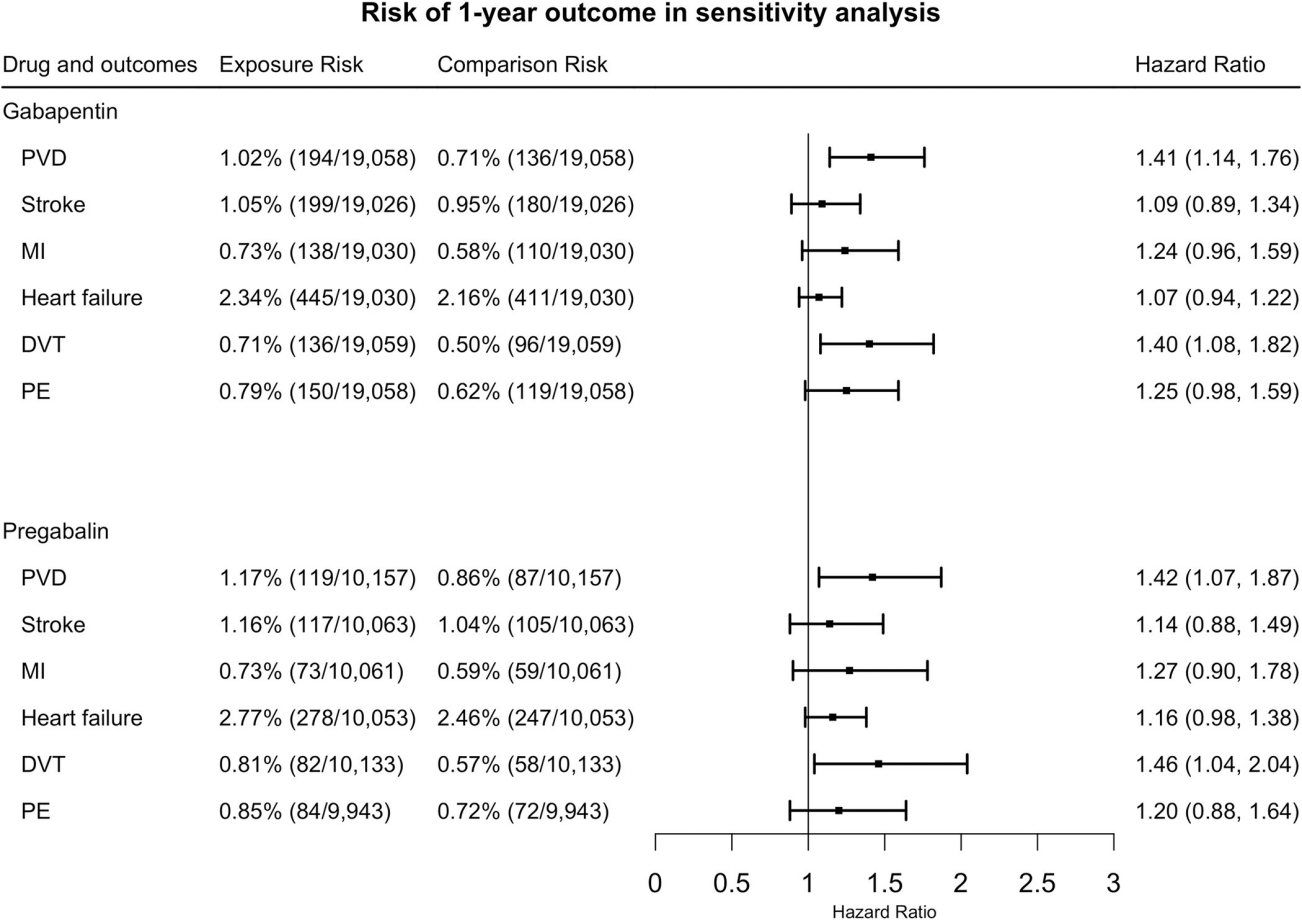
The hazard ratios for 1-year cardiovascular risk between exposures and comparisons after propensity score matching among the subpopulation who had no other possible indications. (PVD: peripheral vascular disease, PE: pulmonary embolism, MI: myocardial infarction, DVT: deep venous thrombosis).

In sensitivity analyses of subpopulations without other possible indications, gabapentin increased the 1-year risk of PVD (HR = 1.41, 95% CI = 1.14–1.76), and DVT (HR = 1.40, 95% CI = 1.08–1.82). Our results indicated no significant association for MI (HR = 1.24, 95% CI = 0.96–1.59), PE (HR = 1.25, 95% CI = 0.98–1.59), stroke (HR = 1.09, 95% CI = 0.89–1.34), or heart failure (HR = 1.07, 95% CI = 0.94–1.22) (Fig 6).

The prescription of pregabalin increased the 1-year risk of PVD (HR = 1.42, 95% CI = 1.07–1.87), and DVT (HR = 1.46, 95% CI = 1.04–2.04). Our results indicate that no significant differences were suggested for PE (HR = 1.20, 95% CI = 0.88–1.64), stroke (HR = 1.14, 95% CI = 0.88–1.49), heart failure (HR = 1.16, 95% CI = 0.98–1.38), or MI (HR = 1.27, 95% CI = 0.90–1.78) (Fig 6).

## Discussion

Using the extensive deidentified TriNetX EHRs database, we reported a moderately increased 5-year risk of several adverse cardiovascular events, including deep venous thrombosis, and pulmonary embolism in patients with fibromyalgia who had a repeated prescription of gabapentin and pregabalin after initial prescription. Patients prescribed gabapentin repeatedly were at a higher 5-year risk of peripheral vascular disease, heart failure, and myocardial infarction. Although other adverse cardiovascular events showed no significant associations in the 5-year follow-up, there was an increased trend in patients prescribed gabapentinoids. At the 1-year follow-up, both the prescription of gabapentin and pregabalin elevated the risk of peripheral vascular disease and pulmonary embolism, and the prescription of gabapentin also elevated the risk of deep venous thrombosis. The sensitivity analyses of the subpopulations that did not have other possible indications showed similar trends to those of the main analysis.

Our previous study [7], along with a recent study involving a Hungarian population [26], demonstrated that the prescription of gabapentin and pregabalin in diabetic neuropathy cohort is associated with an elevated risk of adverse cardiovascular events in long-term follow-up. Similar long-term associations in fibromyalgia patients were revealed in this study. The long-term associations were stronger than the short-term associations for each drug; however, the two study populations (general patients who had a prescription vs. patients who had a repeated prescription after 3 years) with each drug had different characteristics (S3-S6 Tables in S1 File); for example, the average age after matching was younger for general patients than for patients with a repeated prescription. While further confirmation is required to be conducted in other cohorts that use gabapentin and pregabalin, this study underscores the necessity of carefully weighing the risks and benefits of the prescription of gabapentin and pregabalin. Moreover, our results suggest that FDA should consider requiring a warning on the drug label for gabapentin and pregabalin regarding the long-term risk of adverse cardiovascular events.

However, the underlying mechanism for the increased cardiovascular risk associated with gabapentin and pregabalin is unclear. One possible explanation is that both drugs can cause fluid retention [27]. Fluid retention may increase cardiovascular risk [28]. Long-term consumption of gabapentinoids may cause weight gain [29]. Increased body weight can stress the cardiovascular system [30]. In addition, animal studies have shown that gabapentin can reduce blood pressure, heart rate, vascular function, and left ventricular systolic/diastolic function [31–34], potentially leading to adverse cardiovascular events [35–37].

A few limitations of this study are worth mentioning. First of all, the individuals in the TriNetX platform reflects the individuals who had medical encounters with the 60 participating healthcare systems. Whether our findings are generalized should be validated with other data resources and analytics platforms. Second, despite propensity score matching the exposure and comparison cohorts for demographics, risk factors, other possible drug purposes, other related medications, and the socioeconomic status, there are limitations of residual confounding, uncontrolled or unmeasured confounding as well as other potential biases that are common in EHR-based retrospective cohort studies. For example, the prevalence of diabetic neuropathy was still higher in the exposure group compared to the comparison group (2.3% vs. 1.9%), though this difference was not statistically significant. Given the inherent characteristics of EHR data, the TriNetX platform records patient information only at the time of healthcare encounters. Events outside of these encounters, such as insurance details, regional factors, and employment history may not be documented in patient EHRs, and these unmeasured or uncontrolled factors could potentially influence our results. Third, due to the limited sample size, we did not investigate how varying dosages of gabapentin or pregabalin might differentially influence the risk of adverse cardiovascular events. Forth, the duration of drug use is a critical covariate, and we determined exposure to gabapentin and pregabalin using a computerized database. The database indicates only the prescription of medications but does not confirm their actual consumption. EHRs neither provide information on the duration of patients’ drug intake or their adherence.

## Conclusions

Our findings indicate fibromyalgia patients with the prescription of gabapentin and pregabalin were at an elevated risk of various adverse cardiovascular events. The cardiovascular risk data from this study, together with other adverse reactions, patient tolerance, and the effectiveness of pain management should guide healthcare providers in making informed decisions when prescribing these medications within this population.

## Supporting information

S1 File Supporting information. (DOCX)

## Abbreviations

CVDs: Cardiovascular diseases
NSAIDs: Nonselective nonsteroidal anti-inflammatory drugs
CIs: Confidence intervals
HRs: Hazard ratios
SMD: Standardized mean difference
EHRs: Electronic health records
BP: Blood pressure
