# Supplementary material for: Association of adverse cardiovascular events with gabapentin and pregabalin among patients with fibromyalgia
Source: PLoS One. 2024 Jul 26;19(7):e0307515. doi: 10.1371/journal.pone.0307515 (PMC11280525; doi:10.1371/journal.pone.0307515)
Supplement: S1 File — (DOCX) [file pone.0307515.s001.docx]

**Supplementary information**

Association of adverse cardiovascular events with gabapentin and pregabalin among patients with fibromyalgia

**S1 Table**: Outcomes and their standard names, identifiers, and data formats that are used in the TriNetX database

**S2 Table:** Covariates and their standard names, identifiers, and data formats that are used in the TriNetX database

**S3 Table:** Characteristics of fibromyalgia patients with repeated prescription of gabapentin and those with repeated prescription of comparison drugs before and after applying propensity-score matching for pulmonary embolism-related covariates.

**S4 Table:** Characteristics of fibromyalgia patients with repeated prescription of pregabalin and those with repeated prescription of comparison drugs before and after applying propensity-score matching for pulmonary embolism-related covariates.

**S5 Table:** Characteristics of fibromyalgia patients with a prescription of gabapentin and those with a prescription of comparison drugs before and after applying propensity-score matching for pulmonary embolism-related covariates.

**S6 Table:** Characteristics of fibromyalgia patients with a prescription of pregabalin and those with a prescription of comparison drugs before and after applying propensity-score matching for pulmonary embolism-related covariates.

**S1 Table:** Outcomes and their standard names, identifiers, and data formats that are used in the TriNetX database

| **Outcomes** | **Identifier** | **Standard names** | **Data formats** |
| --- | --- | --- | --- |
| Heart failure | ICD-10: I50 | Heart failure | Present/absent |
| Myocardial infarction | ICD-10: I21 | Acute myocardial infarction | Present/absent |
| Peripheral vascular disease | ICD-10: I73.9 | Peripheral vascular diseases, unspecified | Present/absent |
| Stroke | ICD-10: I63 | Cerebral infarction | Present/absent |
| Deep venous thrombosis | ICD-10: I82.40 | Acute embolism and thrombosis of unspecified deep veins of lower extremity | Present/absent |
| Pulmonary embolism | ICD-10: I26 | Pulmonary embolism | Present/absent |

**S2 Table:** Covariates and their standard names, identifiers and data formats that are used in the TriNetX database

| **Covariate** | **Identifier** | **Standard names** | **Data formats** |
| --- | --- | --- | --- |
| Demographics | | | |
| Age at Index | AI | Age at Index | Continuous |
| Female | Demographics: F | Female | Present/absent |
| White | Demographics: 2106-3 | White | Present/absent |
| Black or African American | Demographics: 2054-5 | Black or African American | Present/absent |
| Unknown Race | Demographics: 2131-1 | Unknown Race | Present/absent |
| Hispanic or Latino | Demographics: 2135-2 | Hispanic or Latino | Present/absent |
| Asian | Demographics: 2028-9 | Asian | Present/absent |
| Comorbidities and risk factors | | | |
| Diabetes mellitus | ICD-10: E08-E13 | Diabetes mellitus | Present/absent |
| Irritable bowel syndrome | ICD-10: K58 | Irritable bowel syndrome | Present/absent |
| Chronic fatigue syndrome | ICD-10: R53.82 | Chronic fatigue, unspecified | Present/absent |
| Major depression | ICD-10: F32 | Depressive episode | Present/absent |
| Migraine | ICD-10: G43 | Migraine | Present/absent |
| Panic disorder | ICD-10: F41.0 | Panic disorder (episodic paroxysmal anxiety] | Present/absent |
| Hypertension | ICD-10: I10-I16 | Hypertensive diseases | Present/absent |
| Overweight and obesity | ICD-10: E66 | Overweight and obesity | Present/absent |
| High cholesterol | ICD-10: E78.0 | Pure hypercholesterolemia | Present/absent |
| Obstructive sleep apnea | ICD-10: G47.33 | Obstructive sleep apnea (adult) (pediatric) | Present/absent |
| Long term use of NSAID | ICD-10: Z79.1 | Long term (current) use of non-steroidal anti-inflammatories (NSAID) | Present/absent |
| End stage renal disease | ICD-10: N18.6 | End stage renal disease | Present/absent |
| Adverse socioeconomic determinants of health | ICD-10: Z55-Z65 | Persons with potential health hazards related to socioeconomic and psychosocial circumstances | Present/absent |
| Alcohol abuse | ICD-10: F10.1 | Alcohol abuse | Present/absent |
| Tobacco use | ICD-10: Z72.0 | Tobacco use | Present/absent |
| Pre-existing heart failure | ICD-10: I50 | Heart failure | Present/absent |
| Pre-existing peripheral vascular disease | ICD-10: I73.9 | Peripheral vascular diseases, unspecified | Present/absent |
| Pre-existing stroke | ICD-10: I63 | Cerebral infarction | Present/absent |
| Coronary artery disease | ICD-10: I25.1 | Atherosclerotic heart disease of native coronary artery | Present/absent |
| Hypoglycemia | ICD-10: E16.2 | Hypoglycemia, unspecified | Present/absent |
| Urinary tract infections | ICD-10: N39.0 | Urinary tract infection, site not specified | Present/absent |
| Other possible drug uses | | | |
| Diabetic Neuropathy | ICD-10: E11.40 | Type 2 diabetes mellitus with diabetic neuropathy, unspecified | Present/absent |
| Seizure | ICD-10: G40 | Epilepsy and recurrent seizures | Present/absent |
| Neuropathic pain | ICD-10: M79.2 | Neuralgia and neuritis, unspecified | Present/absent |
| Postherpetic neuralgia | ICD-10: B02.29 | Other postherpetic nervous system involvement | Present/absent |
| Restless leg syndrome | ICD-10: G25.81 | Restless legs syndrome | Present/absent |
| Risk factors of heart failure | | | |
| Pre-existing heart failure | ICD-10: I50 | Heart failure | Present/absent |
| Myocardial infarction | ICD-10: I21 | Acute myocardial infarction | Present/absent |
| Myocarditis | ICD-10: I51.4 | Myocarditis, unspecified | Present/absent |
| Arrhythmias | ICD-10: I49 | Other cardiac arrhythmias | Present/absent |
| Risk factors of myocardial infarction | | | |
| Pre-existing myocardial infarction | ICD-10: I21 | Acute myocardial infarction | Present/absent |
| Sudden cardiac arrest | ICD-10: Z86.74 | Personal history of sudden cardiac arrest | Present/absent |
| Metabolic syndrome | ICD-10: E88.81 | Metabolic syndrome | Present/absent |
| Risk factors of stroke | | | |
| Pre-existing stroke | ICD-10: I63 | Cerebral infarction | Present/absent |
| Atrial fibrillation | ICD-10: I48 | Atrial fibrillation and flutter | Present/absent |
| Arrhythmias | ICD-10: I49 | Other cardiac arrhythmias | Present/absent |
| Depression | ICD-10: F32 | Depressive episode | Present/absent |
| Risk factors of peripheral vascular disease | | | |
| Pre-existing peripheral vascular disease | ICD-10: I73.9 | Peripheral vascular diseases, unspecified | Present/absent |
| Stroke | ICD-10: I63 | Cerebral infarction | Present/absent |
| Myocardial infarction | ICD-10: I21 | Acute myocardial infarction | Present/absent |
| Atherosclerosis | ICD-10: I70 | Atherosclerosis | Present/absent |
| Risk factors of deep venous thrombosis | | | |
| Pre-existing deep venous thrombosis | ICD-10: I82.40 | Acute embolism and thrombosis of unspecified deep veins of lower extremity | Present/absent |
| Inflammatory bowel disease | ICD-10: K51.9 | Ulcerative colitis, unspecified | Present/absent |
| Cancer | ICD-10: C00-D49 | Neoplasms | Present/absent |
| Risk factors of pulmonary embolism | | | |
| Pre-existing pulmonary embolism | ICD-10: I26 | Pulmonary embolism | Present/absent |
| Cancer | ICD-10: C00-D49 | Neoplasms | Present/absent |
| Medications | | | |
| acetaminophen | RxNorm: 161 | acetaminophen | Present/absent |
| hydrocodone | RxNorm: 5489 | hydrocodone | Present/absent |
| oxycodone | RxNorm: 7804 | oxycodone | Present/absent |
| cyclobenzaprine | RxNorm: 21949 | cyclobenzaprine | Present/absent |
| ibuprofen | RxNorm: 5640 | ibuprofen | Present/absent |
| tramadol | RxNorm: 10689 | tramadol | Present/absent |
| naproxen | RxNorm: 7258 | naproxen | Present/absent |
| diclofenac | RxNorm: 3355 | diclofenac | Present/absent |
| codeine | RxNorm: 2670 | codeine | Present/absent |
| amitriptyline | RxNorm: 704 | amitriptyline | Present/absent |
| celecoxib | RxNorm: 140587 | celecoxib | Present/absent |
| venlafaxine | RxNorm: 39786 | venlafaxine | Present/absent |
| nortriptyline | RxNorm: 7531 | nortriptyline | Present/absent |
| pramipexole | RxNorm: 746741 | pramipexole | Present/absent |
| capsaicin | RxNorm: 1992 | capsaicin | Present/absent |
| metformin | RxNorm: 6809 | metformin | Present/absent |
| sulfonylureas | ATC: A10BB | sulfonylureas | Present/absent |
| Insulin | VA: HS501 | Insulin | Present/absent |
| Alpha gulcosidase inhibitors | ATC: A10BF | Alpha gulcosidase inhibitors | Present/absent |
| Dipeptidyl peptidase-4 | ATC: A10BH | Dipeptidyl peptidase-4 | Present/absent |
| Glucagon-like peptide-1 receptor agonists | ATC: A10BJ | Glucagon-like peptide-1 receptor agonists | Present/absent |
| Sodium-glucose transporter 2 inhibitors | ATC: A10BK | Sodium-glucose transporter 2 inhibitors | Present/absent |
| Pioglitazone | RxNorm: 33738 | Pioglitazone | Present/absent |
| Rosiglitazone | RxNorm: 84108 | Rosiglitazone | Present/absent |
| Ace inhibitors | VA: CV800 | Ace inhibitors | Present/absent |
| Beta blocking agents | ATC: C07 | Beta blocking agents | Present/absent |
| Calcium channel blockers | ATC: C08 | Calcium channel blockers | Present/absent |
| Angiotensin ii receptor blockers | ATC: C09C | Angiotensin ii receptor blockers | Present/absent |
| Thiazides/related diuretics | VA: CV701 | Thiazides/related diuretics | Present/absent |
| Alpha blockers/related | VA: CV150 | Alpha blockers/related | Present/absent |
| Clonidine | RxNorm: 2599 | Clonidine | Present/absent |
| Potassium sparing diuretics | VA: CV704 | Potassium sparing diuretics | Present/absent |
| Anticoagulants | VA: BL110 | Anticoagulants | Present/absent |
| nateglinide | RxNorm: 274332 | nateglinide | Present/absent |
| repaglinide | RxNorm: 73044 | repaglinide | Present/absent |

**S3 Table:** Characteristics of fibromyalgia patients with long-term prescription of gabapentin and those with long-term prescription of comparison drugs before and after applying propensity-score matching for pulmonary embolism-related covariates. (SMD: standardized mean differences, *SMD greater than 0.1, a threshold being recommended for declaring imbalance)

|  | Before matching | | | After matching | | |
| --- | --- | --- | --- | --- | --- | --- |
|  | Cohort, No. (%) | | | Cohort, No. (%) | | |
|  | Gabapentin cohort | Comparison cohort | SMD | Gabapentin cohort | Comparison cohort | SMD |
| Cohort size | 19019 | 4412 |  | 4403 | 4403 |  |
| Age at Index | 55.7 ± 14.6 | 53.2 ± 13.8 | 0.17* | 53.4 ± 14.6 | 53.3 ± 13.8 | 0.01 |
| Female | 74.4 | 84.8 | 0.26* | 85.0 | 84.7 | 0.01 |
| White | 68.7 | 76.7 | 0.18* | 76.6 | 76.7 | 0.00 |
| Black or African American | 15.8 | 7.8 | 0.25* | 7.7 | 7.8 | 0.00 |
| Hispanic or Latino | 7.3 | 5.2 | 0.09 | 5.3 | 5.2 | 0.00 |
| Other Race | 2.1 | 1.7 | 0.03 | 1.9 | 1.7 | 0.02 |
| Asian | 1.4 | 0.7 | 0.06 | 0.8 | 0.7 | 0.01 |
| Neoplasms | 37.1 | 25.3 | 0.26* | 24.6 | 25.3 | 0.02 |
| Overweight and obesity | 26.5 | 23.7 | 0.06 | 23.6 | 23.6 | 0.00 |
| Migraine | 15.8 | 17.7 | 0.05 | 17.5 | 17.7 | 0.00 |
| Diabetes mellitus | 26.8 | 16.4 | 0.26* | 16.7 | 16.4 | 0.01 |
| Urinary tract infection, site not specified | 20.0 | 14.2 | 0.15* | 14.8 | 14.2 | 0.02 |
| Obstructive sleep apnea (adult) (pediatric) | 12.9 | 11.6 | 0.04 | 11.0 | 11.6 | 0.02 |
| Irritable bowel syndrome | 8.0 | 9.3 | 0.05 | 8.9 | 9.3 | 0.01 |
| Pain, unspecified | 11.7 | 7.7 | 0.13* | 7.4 | 7.7 | 0.01 |
| Atherosclerotic heart disease of native coronary artery | 12.3 | 6.6 | 0.20* | 7.2 | 6.6 | 0.03 |
| Persons with potential health hazards related to socioeconomic and psychosocial circumstances | 4.3 | 3.6 | 0.04 | 4.2 | 3.6 | 0.03 |
| Chronic fatigue, unspecified | 2.5 | 4.3 | 0.10* | 4.1 | 4.2 | 0.01 |
| Restless legs syndrome | 5.7 | 3.9 | 0.09 | 3.6 | 3.9 | 0.01 |
| Heart failure | 7.4 | 3.4 | 0.18 | 3.4 | 3.4 | 0.00 |
| Panic disorder [episodic paroxysmal anxiety] | 3.6 | 3.0 | 0.04 | 3.2 | 3.0 | 0.01 |
| Tobacco use | 3.8 | 2.9 | 0.05 | 3.2 | 3.0 | 0.01 |
| Neuralgia and neuritis, unspecified | 6.6 | 2.2 | 0.21* | 2.6 | 2.2 | 0.03 |
| Type 2 diabetes mellitus with diabetic neuropathy, unspecified | 6.0 | 1.9 | 0.21* | 2.3 | 1.9 | 0.03 |
| Epilepsy and recurrent seizures | 3.1 | 2.0 | 0.07 | 2.1 | 2.0 | 0.01 |
| Long term (current) use of non-steroidal anti-inflammatories (NSAID) | 1.6 | 1.7 | 0.01 | 1.9 | 1.7 | 0.01 |
| Peripheral vascular disease, unspecified | 4.8 | 1.8 | 0.17* | 1.8 | 1.8 | 0.01 |
| Cerebral infarction | 3.9 | 1.8 | 0.12* | 1.8 | 1.8 | 0.01 |
| Alcohol abuse | 4.0 | 1.2 | 0.18* | 1.2 | 1.2 | 0.00 |
| Pulmonary embolism | 2.3 | 0.9 | 0.11* | 1.0 | 0.9 | 0.01 |
| Hypoglycemia, unspecified | 1.9 | 1.0 | 0.08 | 1.0 | 1.0 | 0.00 |
| End stage renal disease | 1.3 | 0.3 | 0.11* | 0.2 | 0.3 | 0.01 |
| Other postherpetic nervous system involvement | 0.7 | 0.2 | 0.06 | 0.2 | 0.3 | 0.00 |
| acetaminophen | 63.7 | 44.7 | 0.39* | 43.3 | 44.8 | 0.03 |
| hydrocodone | 39.3 | 26.3 | 0.28* | 25.1 | 26.4 | 0.03 |
| BETA BLOCKING AGENTS | 32.0 | 22.2 | 0.22* | 21.9 | 22.3 | 0.01 |
| cyclobenzaprine | 27.9 | 20.1 | 0.18* | 20.2 | 20.2 | 0.00 |
| oxycodone | 33.4 | 20.1 | 0.30* | 20.1 | 20.1 | 0.00 |
| tramadol | 29.7 | 18.9 | 0.26* | 18.7 | 18.9 | 0.01 |
| ibuprofen | 29.2 | 18.1 | 0.26* | 17.0 | 18.1 | 0.03 |
| THIAZIDES/RELATED DIURETICS | 23.4 | 16.5 | 0.17* | 16.6 | 16.5 | 0.00 |
| ANTICOAGULANTS | 26.7 | 14.7 | 0.30* | 15.2 | 14.8 | 0.01 |
| ACE INHIBITORS | 24.9 | 14.2 | 0.27* | 14.7 | 14.3 | 0.01 |
| CALCIUM CHANNEL BLOCKERS | 20.7 | 12.9 | 0.21* | 12.7 | 12.9 | 0.00 |
| naproxen | 18.6 | 12.2 | 0.18* | 11.1 | 12.2 | 0.03 |
| diclofenac | 15.4 | 11.3 | 0.12* | 10.9 | 11.3 | 0.01 |
| codeine | 17.7 | 10.1 | 0.22* | 10.3 | 10.1 | 0.01 |
| ANGIOTENSIN II RECEPTOR BLOCKERS (ARBs), PLAIN | 13.4 | 10.2 | 0.10* | 10.0 | 10.2 | 0.00 |
| amitriptyline | 9.9 | 9.2 | 0.02 | 9.4 | 9.2 | 0.01 |
| metformin | 14.6 | 8.6 | 0.19* | 9.0 | 8.6 | 0.02 |
| INSULIN | 14.4 | 6.4 | 0.26* | 6.8 | 6.4 | 0.02 |
| venlafaxine | 6.6 | 5.7 | 0.04 | 5.7 | 5.7 | 0.00 |
| celecoxib | 7.8 | 5.3 | 0.10* | 5.4 | 5.3 | 0.00 |
| POTASSIUM SPARING/COMBINATIONS DIURETICS | 6.5 | 4.6 | 0.08 | 4.6 | 4.6 | 0.00 |
| ALPHA BLOCKERS/RELATED | 6.9 | 3.4 | 0.16 | 3.8 | 3.4 | 0.02 |
| nortriptyline | 3.7 | 3.1 | 0.03 | 2.6 | 3.1 | 0.03 |
| Sulfonylureas | 6.8 | 2.5 | 0.21 | 2.6 | 2.5 | 0.01 |
| Dipeptidyl peptidase 4 (DPP-4) inhibitors | 2.8 | 1.4 | 0.10 | 1.7 | 1.4 | 0.02 |
| clonidine | 4.3 | 1.6 | 0.16* | 1.7 | 1.6 | 0.00 |
| Glucagon-like peptide-1 (GLP-1) analogues | 1.8 | 1.4 | 0.03 | 1.6 | 1.4 | 0.02 |
| pramipexole | 1.4 | 1.1 | 0.03 | 1.3 | 1.1 | 0.02 |
| Sodium-glucose co-transporter 2 (SGLT2) inhibitors | 0.7 | 0.7 | 0.00 | 0.8 | 0.7 | 0.02 |
| pioglitazone | 1.6 | 0.7 | 0.09 | 0.8 | 0.7 | 0.01 |
| capsaicin | 1.5 | 0.6 | 0.09 | 0.5 | 0.6 | 0.00 |
| Alpha glucosidase inhibitors | 0.1 | 0.2 | 0.03 | 0.2 | 0.2 | 0.00 |
| rosiglitazone | 0.3 | 0.2 | 0.01 | 0.2 | 0.2 | 0.00 |
| nateglinide | 0.1 | 0.2 | 0.03 | 0.2 | 0.2 | 0.00 |
| repaglinide | 0.3 | 0.2 | 0.02 | 0.2 | 0.2 | 0.00 |

**Table S4:** Characteristics of fibromyalgia patients with long-term prescription of pregabalin and those with long-term prescription of comparison drugs before and after applying propensity-score matching for pulmonary embolism-related covariates. (SMD: standardized mean differences, *SMD greater than 0.1, a threshold being recommended for declaring imbalance)

|  | Before matching | | | After matching | | |
| --- | --- | --- | --- | --- | --- | --- |
|  | Cohort, No. (%) | | | Cohort, No. (%) | | |
|  | Pregabalin cohort | Comparison cohort | SMD | Pregabalin cohort | Comparison cohort | SMD |
| Cohort size | 2458 | 4412 |  | 2286 | 2286 |  |
| Age at Index | 53.7 ± 13.5 | 53.2 ± 13.8 | 0.04 | 53.6 ± 13.6 | 53.8 ± 13.8 | 0.02 |
| Female | 82.0 | 84.8 | 0.07 | 83.0 | 83.1 | 0.00 |
| White | 74.5 | 76.7 | 0.05 | 75.2 | 75.7 | 0.01 |
| Black or African American | 11.8 | 7.8 | 0.14* | 10.7 | 10.2 | 0.02 |
| Hispanic or Latino | 5.4 | 5.2 | 0.01 | 5.4 | 5.7 | 0.01 |
| Other Race | 1.6 | 1.7 | 0.01 | 1.6 | 1.6 | 0.00 |
| Asian | 1.0 | 0.7 | 0.03 | 1.0 | 0.9 | 0.01 |
| Neoplasms | 24.4 | 25.3 | 0.02 | 23.6 | 23.7 | 0.00 |
| Overweight and obesity | 22.4 | 23.7 | 0.03 | 21.6 | 22.2 | 0.01 |
| Diabetes mellitus | 21.8 | 16.4 | 0.14* | 19.4 | 19.6 | 0.00 |
| Migraine | 17.3 | 17.7 | 0.01 | 17.8 | 17.8 | 0.00 |
| Urinary tract infection, site not specified | 12.8 | 14.2 | 0.04 | 12.9 | 12.7 | 0.01 |
| Obstructive sleep apnea (adult) (pediatric) | 10.4 | 11.6 | 0.04 | 10.2 | 10.7 | 0.02 |
| Irritable bowel syndrome | 8.5 | 9.3 | 0.03 | 8.6 | 8.9 | 0.01 |
| Pain, unspecified | 7.8 | 7.7 | 0.00 | 7.3 | 8.0 | 0.02 |
| Atherosclerotic heart disease of native coronary artery | 7.3 | 6.6 | 0.03 | 6.8 | 6.9 | 0.00 |
| Restless legs syndrome | 4.6 | 3.9 | 0.04 | 4.3 | 4.4 | 0.01 |
| Heart failure | 4.6 | 3.4 | 0.06 | 4.2 | 4.4 | 0.01 |
| Chronic fatigue, unspecified | 3.2 | 4.3 | 0.06 | 3.4 | 3.0 | 0.02 |
| Tobacco use | 3.4 | 2.9 | 0.03 | 3.4 | 3.0 | 0.02 |
| Neuralgia and neuritis, unspecified | 4.9 | 2.2 | 0.14* | 3.4 | 3.6 | 0.01 |
| Type 2 diabetes mellitus with diabetic neuropathy, unspecified | 4.9 | 1.9 | 0.17* | 3.2 | 3.3 | 0.01 |
| Panic disorder [episodic paroxysmal anxiety] | 3.2 | 3.0 | 0.01 | 2.9 | 3.3 | 0.02 |
| Epilepsy and recurrent seizures | 2.8 | 2.0 | 0.06 | 2.7 | 2.8 | 0.01 |
| Peripheral vascular disease, unspecified | 2.7 | 1.8 | 0.06 | 2.4 | 2.1 | 0.01 |
| Persons with potential health hazards related to socioeconomic and psychosocial circumstances | 2.2 | 3.6 | 0.08 | 2.3 | 2.3 | 0.00 |
| Long term (current) use of non-steroidal anti-inflammatories (NSAID) | 2.2 | 1.7 | 0.03 | 1.8 | 2.0 | 0.01 |
| Cerebral infarction | 2.2 | 1.8 | 0.02 | 1.8 | 2.2 | 0.02 |
| Alcohol abuse | 2.0 | 1.2 | 0.06 | 1.6 | 1.9 | 0.02 |
| Pulmonary embolism | 1.3 | 0.9 | 0.04 | 1.3 | 1.1 | 0.02 |
| Hypoglycemia, unspecified | 1.1 | 1.0 | 0.01 | 1.0 | 1.1 | 0.02 |
| End stage renal disease | 0.5 | 0.3 | 0.04 | 0.4 | 0.4 | 0.00 |
| Other postherpetic nervous system involvement | 0.4 | 0.2 | 0.03 | 0.4 | 0.4 | 0.00 |
| acetaminophen | 50.5 | 44.7 | 0.12* | 48.8 | 50.0 | 0.02 |
| hydrocodone | 29.0 | 26.3 | 0.06 | 28.3 | 29.0 | 0.01 |
| oxycodone | 29.0 | 20.1 | 0.21* | 26.3 | 26.2 | 0.00 |
| BETA BLOCKING AGENTS | 22.5 | 22.2 | 0.01 | 22.0 | 22.5 | 0.01 |
| tramadol | 21.0 | 18.9 | 0.05 | 20.0 | 20.8 | 0.02 |
| cyclobenzaprine | 16.8 | 20.1 | 0.08 | 17.5 | 17.0 | 0.02 |
| ANTICOAGULANTS | 18.3 | 14.7 | 0.10* | 17.4 | 17.3 | 0.00 |
| ibuprofen | 15.1 | 18.1 | 0.08 | 15.4 | 15.4 | 0.00 |
| ACE INHIBITORS | 13.8 | 14.2 | 0.01 | 13.6 | 13.7 | 0.00 |
| THIAZIDES/RELATED DIURETICS | 13.2 | 16.5 | 0.09 | 13.3 | 13.4 | 0.00 |
| CALCIUM CHANNEL BLOCKERS | 12.4 | 12.9 | 0.01 | 12.4 | 12.3 | 0.00 |
| codeine | 9.1 | 10.1 | 0.03 | 9.1 | 9.4 | 0.01 |
| amitriptyline | 9.2 | 9.2 | 0.00 | 8.9 | 8.7 | 0.01 |
| diclofenac | 8.5 | 11.3 | 0.09 | 8.7 | 9.0 | 0.01 |
| naproxen | 8.3 | 12.2 | 0.13* | 8.7 | 8.9 | 0.01 |
| INSULIN | 10.1 | 6.4 | 0.14* | 8.6 | 8.7 | 0.00 |
| metformin | 8.8 | 8.6 | 0.01 | 8.5 | 8.3 | 0.01 |
| ANGIOTENSIN II RECEPTOR BLOCKERS (ARBs), PLAIN | 7.7 | 10.2 | 0.09 | 7.8 | 7.7 | 0.00 |
| celecoxib | 8.4 | 5.3 | 0.12* | 7.3 | 7.3 | 0.00 |
| venlafaxine | 6.3 | 5.7 | 0.02 | 6.1 | 5.9 | 0.01 |
| POTASSIUM SPARING/COMBINATIONS DIURETICS | 4.1 | 4.6 | 0.03 | 4.2 | 4.3 | 0.00 |
| Sulfonylureas | 3.2 | 2.5 | 0.04 | 3.2 | 3.2 | 0.00 |
| nortriptyline | 3.1 | 3.1 | 0.00 | 3.1 | 2.9 | 0.01 |
| ALPHA BLOCKERS/RELATED | 3.1 | 3.4 | 0.02 | 2.9 | 3.5 | 0.03 |
| clonidine | 2.5 | 1.6 | 0.06 | 2.1 | 1.8 | 0.02 |
| Dipeptidyl peptidase 4 (DPP-4) inhibitors | 1.9 | 1.4 | 0.03 | 1.8 | 2.0 | 0.01 |
| Glucagon-like peptide-1 (GLP-1) analogues | 1.5 | 1.4 | 0.01 | 1.6 | 1.4 | 0.02 |
| pramipexole | 1.3 | 1.1 | 0.02 | 1.3 | 1.4 | 0.02 |
| pioglitazone | 0.7 | 0.7 | 0.00 | 0.7 | 0.7 | 0.01 |
| Sodium-glucose co-transporter 2 (SGLT2) inhibitors | 0.5 | 0.7 | 0.02 | 0.5 | 0.7 | 0.02 |
| capsaicin | 0.4 | 0.6 | 0.02 | 0.4 | 0.5 | 0.01 |
| nateglinide | 0.4 | 0.2 | 0.03 | 0.4 | 0.4 | 0.00 |
| repaglinide | 0.4 | 0.2 | 0.03 | 0.4 | 0.4 | 0.00 |
| Alpha glucosidase inhibitors | 0.4 | 0.2 | 0.03 | 0.0 | 0.4 | 0.09 |
| rosiglitazone | 0.0 | 0.2 | 0.07 | 0.0 | 0.0 |  |

**S5 Table:** Characteristics of fibromyalgia patients with a prescription of gabapentin and those with a prescription of comparison drugs before and after applying propensity-score matching for pulmonary embolism-related covariates. (SMD: standardized mean differences, *SMD greater than 0.1, a threshold being recommended for declaring imbalance)

|  | Before matching | | | After matching | | |
| --- | --- | --- | --- | --- | --- | --- |
|  | Cohort, No. (%) | | | Cohort, No. (%) | | |
|  | Gabapentin cohort | Comparison cohort | SMD | Gabapentin cohort | Comparison cohort | SMD |
| Cohort size | 69281 | 22589 |  | 22580 | 22580 |  |
| Age at Index | 54 ± 15.8 | 51.2 ± 15.2 | 0.19* | 51.1 ± 15.7 | 51.2 ± 15.2 | 0.00 |
| Female | 74.6 | 85.1 | 0.26* | 85.1 | 85.1 | 0.00 |
| White | 69.9 | 73.9 | 0.09 | 73.8 | 73.8 | 0.00 |
| Black or African American | 13.3 | 8.2 | 0.17* | 8.1 | 8.2 | 0.00 |
| Hispanic or Latino | 7.5 | 6.5 | 0.04 | 6.7 | 6.6 | 0.00 |
| Other Race | 2.4 | 2.6 | 0.01 | 2.6 | 2.6 | 0.00 |
| Asian | 1.3 | 1.0 | 0.03 | 1.0 | 1.0 | 0.00 |
| Neoplasms | 32.6 | 22.0 | 0.24* | 22.0 | 22.0 | 0.00 |
| Overweight and obesity | 23.0 | 19.3 | 0.09 | 19.1 | 19.3 | 0.00 |
| Migraine | 14.5 | 16.8 | 0.06 | 16.4 | 16.8 | 0.01 |
| Diabetes mellitus | 22.1 | 13.7 | 0.22* | 13.5 | 13.7 | 0.01 |
| Urinary tract infection, site not specified | 16.9 | 12.0 | 0.14* | 11.7 | 12.0 | 0.01 |
| Obstructive sleep apnea (adult) (pediatric) | 10.7 | 8.7 | 0.07 | 8.4 | 8.7 | 0.01 |
| Irritable bowel syndrome | 7.1 | 8.3 | 0.05 | 8.1 | 8.3 | 0.01 |
| Pain, unspecified | 10.4 | 7.8 | 0.09 | 7.9 | 7.8 | 0.00 |
| Atherosclerotic heart disease of native coronary artery | 11.1 | 6.4 | 0.17* | 6.5 | 6.4 | 0.01 |
| Chronic fatigue, unspecified | 2.5 | 4.2 | 0.09 | 3.9 | 4.1 | 0.01 |
| Heart failure | 7.0 | 3.6 | 0.16* | 3.5 | 3.6 | 0.00 |
| Persons with potential health hazards related to socioeconomic and psychosocial circumstances | 3.9 | 3.4 | 0.03 | 3.5 | 3.3 | 0.01 |
| Restless legs syndrome | 4.5 | 3.3 | 0.06 | 3.4 | 3.3 | 0.01 |
| Panic disorder [episodic paroxysmal anxiety] | 3.1 | 3.1 | 0.00 | 3.1 | 3.1 | 0.00 |
| Tobacco use | 3.4 | 2.6 | 0.05 | 2.7 | 2.6 | 0.00 |
| Neuralgia and neuritis, unspecified | 5.7 | 2.6 | 0.16* | 2.6 | 2.6 | 0.00 |
| Epilepsy and recurrent seizures | 2.9 | 2.1 | 0.05 | 2.1 | 2.1 | 0.00 |
| Cerebral infarction | 3.5 | 1.9 | 0.10* | 1.9 | 1.9 | 0.00 |
| Peripheral vascular disease, unspecified | 4.1 | 1.8 | 0.14* | 1.7 | 1.8 | 0.00 |
| Type 2 diabetes mellitus with diabetic neuropathy, unspecified | 4.4 | 1.5 | 0.17* | 1.6 | 1.5 | 0.01 |
| Alcohol abuse | 3.6 | 1.6 | 0.13* | 1.5 | 1.6 | 0.01 |
| Long term (current) use of non-steroidal anti-inflammatories (NSAID) | 1.6 | 1.2 | 0.04 | 1.1 | 1.2 | 0.00 |
| Pulmonary embolism | 2.1 | 1.2 | 0.07 | 1.1 | 1.2 | 0.01 |
| Hypoglycemia, unspecified | 1.7 | 0.9 | 0.07 | 1.0 | 0.9 | 0.01 |
| End stage renal disease | 1.3 | 0.3 | 0.12* | 0.3 | 0.3 | 0.01 |
| Other postherpetic nervous system involvement | 0.7 | 0.2 | 0.07 | 0.3 | 0.2 | 0.01 |
| acetaminophen | 62.6 | 46.6 | 0.33* | 46.4 | 46.7 | 0.01 |
| hydrocodone | 35.8 | 25.6 | 0.22* | 25.7 | 25.6 | 0.00 |
| oxycodone | 34.4 | 21.9 | 0.28* | 21.7 | 21.9 | 0.01 |
| BETA BLOCKING AGENTS | 29.7 | 21.4 | 0.19* | 20.8 | 21.4 | 0.01 |
| cyclobenzaprine | 24.9 | 19.7 | 0.13* | 19.8 | 19.7 | 0.00 |
| ibuprofen | 28.0 | 19.4 | 0.20* | 19.3 | 19.5 | 0.00 |
| tramadol | 26.9 | 18.3 | 0.21* | 18.4 | 18.3 | 0.00 |
| ANTICOAGULANTS | 26.8 | 16.3 | 0.26* | 15.7 | 16.3 | 0.02 |
| THIAZIDES/RELATED DIURETICS | 19.6 | 14.1 | 0.15* | 13.9 | 14.1 | 0.00 |
| ACE INHIBITORS | 21.0 | 13.5 | 0.20* | 13.3 | 13.5 | 0.00 |
| CALCIUM CHANNEL BLOCKERS | 18.4 | 12.3 | 0.17* | 11.9 | 12.3 | 0.01 |
| naproxen | 17.1 | 11.9 | 0.15* | 11.9 | 11.9 | 0.00 |
| diclofenac | 13.5 | 10.5 | 0.09 | 10.4 | 10.5 | 0.00 |
| codeine | 15.1 | 9.5 | 0.17* | 9.2 | 9.5 | 0.01 |
| amitriptyline | 9.5 | 9.1 | 0.01 | 9.2 | 9.1 | 0.00 |
| ANGIOTENSIN II RECEPTOR BLOCKERS (ARBs), PLAIN | 11.3 | 9.0 | 0.08 | 8.8 | 9.0 | 0.01 |
| metformin | 11.2 | 7.1 | 0.14* | 7.1 | 7.1 | 0.00 |
| INSULIN | 12.9 | 6.7 | 0.21* | 6.6 | 6.7 | 0.00 |
| celecoxib | 7.1 | 5.0 | 0.09 | 5.0 | 5.0 | 0.00 |
| venlafaxine | 6.0 | 4.6 | 0.06 | 4.7 | 4.6 | 0.00 |
| POTASSIUM SPARING/COMBINATIONS DIURETICS | 5.9 | 4.3 | 0.07 | 4.0 | 4.3 | 0.02 |
| nortriptyline | 3.5 | 3.1 | 0.02 | 3.2 | 3.1 | 0.01 |
| ALPHA BLOCKERS/RELATED | 6.0 | 3.1 | 0.14* | 3.1 | 3.1 | 0.00 |
| clonidine | 4.2 | 2.3 | 0.11* | 2.4 | 2.3 | 0.01 |
| Sulfonylureas | 5.1 | 2.3 | 0.15* | 2.3 | 2.3 | 0.00 |
| Dipeptidyl peptidase 4 (DPP-4) inhibitors | 2.1 | 1.3 | 0.07 | 1.3 | 1.3 | 0.00 |
| pramipexole | 1.2 | 1.1 | 0.01 | 1.2 | 1.1 | 0.00 |
| Glucagon-like peptide-1 (GLP-1) analogues | 1.1 | 0.8 | 0.03 | 0.8 | 0.8 | 0.00 |
| pioglitazone | 1.1 | 0.5 | 0.07 | 0.6 | 0.5 | 0.01 |
| capsaicin | 1.3 | 0.6 | 0.07 | 0.6 | 0.6 | 0.00 |
| Sodium-glucose co-transporter 2 (SGLT2) inhibitors | 0.5 | 0.5 | 0.01 | 0.5 | 0.5 | 0.00 |
| rosiglitazone | 0.2 | 0.1 | 0.03 | 0.1 | 0.1 | 0.00 |
| repaglinide | 0.2 | 0.1 | 0.03 | 0.1 | 0.1 | 0.01 |
| Alpha glucosidase inhibitors | 0.1 | 0.0 | 0.02 | 0.0 | 0.0 | 0.00 |
| nateglinide | 0.1 | 0.0 | 0.02 | 0.0 | 0.0 | 0.00 |

**S6 Table:** Characteristics of fibromyalgia patients with a prescription of pregabalin and those with a prescription of comparison drugs before and after applying propensity-score matching for pulmonary embolism-related covariates. (SMD: standardized mean differences, *SMD greater than 0.1, a threshold being recommended for declaring imbalance)

|  | Before matching | | | After matching | | |
| --- | --- | --- | --- | --- | --- | --- |
|  | Cohort, No. (%) | | | Cohort, No. (%) | | |
|  | Pregabalin cohort | Comparison cohort | SMD | Pregabalin cohort | Comparison cohort | SMD |
| Cohort size | 13304 | 21954 |  | 12444 | 12444 |  |
| Age at Index | 54 ± 15.8 | 51.2 ± 15.2 | 0.06 | 51.1 ± 15.7 | 51.2 ± 15.2 | 0.00 |
| Female | 80.6 | 84.8 | 0.11* | 82.0 | 81.8 | 0.00 |
| White | 73.7 | 73.6 | 0.00 | 73.5 | 72.8 | 0.01 |
| Black or African American | 10.1 | 8.3 | 0.06 | 9.8 | 9.7 | 0.00 |
| Hispanic or Latino | 6.8 | 6.4 | 0.02 | 6.9 | 6.8 | 0.00 |
| Other Race | 2.2 | 2.4 | 0.01 | 2.3 | 2.2 | 0.00 |
| Asian | 1.0 | 1.0 | 0.00 | 1.0 | 1.1 | 0.00 |
| Neoplasms | 21.9 | 22.2 | 0.01 | 21.5 | 21.7 | 0.00 |
| Overweight and obesity | 19.4 | 19.5 | 0.00 | 19.0 | 18.8 | 0.00 |
| Diabetes mellitus | 18.5 | 13.8 | 0.13* | 16.8 | 16.9 | 0.00 |
| Migraine | 14.6 | 16.8 | 0.06 | 15.0 | 14.8 | 0.01 |
| Urinary tract infection, site not specified | 11.6 | 12.2 | 0.02 | 11.6 | 11.6 | 0.00 |
| Obstructive sleep apnea (adult) (pediatric) | 9.4 | 8.7 | 0.02 | 9.0 | 9.2 | 0.01 |
| Irritable bowel syndrome | 7.3 | 8.3 | 0.04 | 7.5 | 7.3 | 0.01 |
| Pain, unspecified | 7.5 | 7.9 | 0.01 | 7.4 | 7.4 | 0.00 |
| Atherosclerotic heart disease of native coronary artery | 7.9 | 6.4 | 0.06 | 7.2 | 7.4 | 0.01 |
| Heart failure | 5.1 | 3.6 | 0.08 | 4.5 | 4.6 | 0.00 |
| Neuralgia and neuritis, unspecified | 4.2 | 2.6 | 0.09 | 3.6 | 3.6 | 0.00 |
| Restless legs syndrome | 3.5 | 3.2 | 0.02 | 3.4 | 3.3 | 0.01 |
| Chronic fatigue, unspecified | 2.7 | 4.0 | 0.07 | 2.8 | 2.9 | 0.01 |
| Tobacco use | 2.8 | 2.7 | 0.01 | 2.8 | 2.8 | 0.00 |
| Epilepsy and recurrent seizures | 2.8 | 2.1 | 0.05 | 2.7 | 2.7 | 0.00 |
| Panic disorder [episodic paroxysmal anxiety] | 2.5 | 3.1 | 0.04 | 2.6 | 2.5 | 0.01 |
| Persons with potential health hazards related to socioeconomic and psychosocial circumstances | 2.5 | 3.4 | 0.06 | 2.6 | 2.6 | 0.00 |
| Type 2 diabetes mellitus with diabetic neuropathy, unspecified | 3.5 | 1.5 | 0.13* | 2.3 | 2.5 | 0.01 |
| Peripheral vascular disease, unspecified | 2.6 | 1.8 | 0.05 | 2.3 | 2.3 | 0.00 |
| Cerebral infarction | 2.5 | 2.0 | 0.04 | 2.3 | 2.3 | 0.00 |
| Alcohol abuse | 2.0 | 1.6 | 0.03 | 1.8 | 1.9 | 0.00 |
| Long term (current) use of non-steroidal anti-inflammatories (NSAID) | 1.7 | 1.2 | 0.04 | 1.5 | 1.5 | 0.00 |
| Pulmonary embolism | 1.6 | 1.2 | 0.04 | 1.5 | 1.5 | 0.00 |
| Hypoglycemia, unspecified | 1.1 | 0.9 | 0.02 | 1.1 | 1.1 | 0.00 |
| End stage renal disease | 0.9 | 0.3 | 0.08 | 0.5 | 0.5 | 0.01 |
| Other postherpetic nervous system involvement | 0.5 | 0.2 | 0.04 | 0.4 | 0.4 | 0.00 |
| acetaminophen | 55.2 | 46.4 | 0.18* | 52.7 | 52.7 | 0.00 |
| oxycodone | 31.5 | 21.7 | 0.22* | 28.5 | 28.5 | 0.00 |
| hydrocodone | 28.3 | 25.4 | 0.07 | 27.6 | 27.6 | 0.00 |
| BETA BLOCKING AGENTS | 23.5 | 21.4 | 0.05 | 22.5 | 22.5 | 0.00 |
| tramadol | 21.4 | 18.1 | 0.08 | 20.3 | 20.4 | 0.00 |
| ANTICOAGULANTS | 21.8 | 16.3 | 0.14* | 20.2 | 20.3 | 0.00 |
| ibuprofen | 17.4 | 19.4 | 0.05 | 17.7 | 18.0 | 0.01 |
| cyclobenzaprine | 16.9 | 19.6 | 0.07 | 17.4 | 17.8 | 0.01 |
| ACE INHIBITORS | 13.7 | 13.4 | 0.01 | 13.5 | 13.7 | 0.00 |
| CALCIUM CHANNEL BLOCKERS | 12.8 | 12.3 | 0.01 | 12.5 | 12.5 | 0.00 |
| THIAZIDES/RELATED DIURETICS | 12.2 | 14.1 | 0.05 | 12.4 | 12.6 | 0.01 |
| naproxen | 9.7 | 11.9 | 0.07 | 9.9 | 10.3 | 0.01 |
| diclofenac | 9.1 | 10.6 | 0.05 | 9.3 | 9.6 | 0.01 |
| INSULIN | 10.5 | 6.7 | 0.14* | 9.1 | 9.1 | 0.00 |
| amitriptyline | 8.8 | 9.0 | 0.01 | 8.9 | 8.7 | 0.01 |
| codeine | 8.7 | 9.5 | 0.03 | 8.7 | 8.3 | 0.01 |
| ANGIOTENSIN II RECEPTOR BLOCKERS (ARBs), PLAIN | 7.5 | 9.0 | 0.05 | 7.6 | 8.0 | 0.02 |
| metformin | 7.7 | 7.1 | 0.02 | 7.5 | 7.8 | 0.01 |
| celecoxib | 10.1 | 5.0 | 0.19* | 7.1 | 7.7 | 0.02 |
| venlafaxine | 6.0 | 4.6 | 0.07 | 5.6 | 5.6 | 0.00 |
| POTASSIUM SPARING/COMBINATIONS DIURETICS | 4.0 | 4.3 | 0.01 | 4.0 | 4.3 | 0.02 |
| ALPHA BLOCKERS/RELATED | 3.8 | 3.1 | 0.03 | 3.5 | 3.5 | 0.00 |
| nortriptyline | 3.2 | 3.1 | 0.01 | 3.2 | 3.4 | 0.01 |
| Sulfonylureas | 3.2 | 2.3 | 0.06 | 3.0 | 2.9 | 0.00 |
| clonidine | 3.0 | 2.3 | 0.04 | 2.7 | 2.8 | 0.01 |
| Dipeptidyl peptidase 4 (DPP-4) inhibitors | 1.5 | 1.2 | 0.02 | 1.4 | 1.4 | 0.00 |
| pramipexole | 1.4 | 1.1 | 0.02 | 1.3 | 1.4 | 0.00 |
| Glucagon-like peptide-1 (GLP-1) analogues | 1.0 | 0.8 | 0.02 | 1.0 | 1.0 | 0.00 |
| capsaicin | 0.6 | 0.6 | 0.00 | 0.6 | 0.5 | 0.01 |
| pioglitazone | 0.7 | 0.5 | 0.02 | 0.6 | 0.7 | 0.01 |
| Sodium-glucose co-transporter 2 (SGLT2) inhibitors | 0.4 | 0.4 | 0.01 | 0.4 | 0.3 | 0.01 |
| repaglinide | 0.1 | 0.1 | 0.00 | 0.1 | 0.1 | 0.00 |
| Alpha glucosidase inhibitors | 0.1 | 0.0 | 0.01 | 0.1 | 0.1 | 0.00 |
| rosiglitazone | 0.1 | 0.1 | 0.00 | 0.1 | 0.1 | 0.00 |
| nateglinide | 0.1 | 0.0 | 0.01 | 0.1 | 0.1 | 0.00 |
